# Metagenomic Characterization of the Microbiome and Resistome of Retail Ground Beef Products

**DOI:** 10.3389/fmicb.2020.541972

**Published:** 2020-11-06

**Authors:** Enrique Doster, Kevin M. Thomas, Maggie D. Weinroth, Jennifer K. Parker, Kathryn K. Crone, Terrance M. Arthur, John W. Schmidt, Tommy L. Wheeler, Keith E. Belk, Paul S. Morley

**Affiliations:** ^1^Texas A&M University, VERO Program, Canyon, TX, United States; ^2^Department of Microbiology, Immunology and Pathology, College of Veterinary Medicine and Biomedical Sciences, Colorado State University, Fort Collins, CO, United States; ^3^Department of Veterinary Population Medicine, College of Veterinary Medicine, University of Minnesota, Saint Paul, MN, United States; ^4^Department of Animal Sciences, College of Agricultural Sciences, Colorado State University, Fort Collins, CO, United States; ^5^Department of Clinical Sciences, College of Veterinary Medicine and Biomedical Sciences, Colorado State University, Fort Collins, CO, United States; ^6^U.S. Meat Animal Research Center, Agricultural Research Service, United States Department of Agriculture, Clay Center, NE, United States

**Keywords:** metagenomic sequencing, ground beef, raised without antibiotics, antimicrobial resistance, resistome, microbiome

## Abstract

Ground beef can be a reservoir for a variety of bacteria, including spoilage organisms, and pathogenic foodborne bacteria. These bacteria can exhibit antimicrobial resistance (AMR) which is a public health concern if resistance in pathogens leads to treatment failure in humans. Culture-dependent techniques are commonly used to study individual bacterial species, but these techniques are unable to describe the whole community of microbial species (microbiome) and the profile of AMR genes they carry (resistome), which is critical for getting a holistic perspective of AMR. The objective of this study was to characterize the microbiome and resistome of retail ground beef products labeled as coming from conventional or raised without antibiotics (RWA) production systems. Sixteen ground beef products were purchased from 6 retail grocery outlets in Fort Collins, CO, half of which were labeled as produced from cattle raised conventionally and half of products were from RWA production. Total DNA was extracted and isolated from each sample and subjected to 16S rRNA amplicon sequencing for microbiome characterization and target-enriched shotgun sequencing to characterize the resistome. Differences in the microbiome and resistome of RWA and conventional ground beef were analyzed using the R programming software. Our results suggest that the resistome and microbiome of retail ground beef products with RWA packaging labels do not differ from products that do not carry claims regarding antimicrobial drug exposures during cattle production. The resistome predominantly consisted of tetracycline resistance making up more than 90% of reads mapped to resistance gene accessions in our samples. Firmicutes and Proteobacteria predominated in the microbiome of all samples (69.6% and 29.0%, respectively), but Proteobacteria composed a higher proportion in ground beef from conventionally raised cattle. In addition, our results suggest that product management, such as packaging type, could exert a stronger influence on the microbiome than the resistome in consumer-ready products. Metagenomic analyses of ground beef is a promising tool to investigate community-wide shifts in retail ground beef. Importantly, however, results from metagenomic sequencing must be carefully considered in parallel with traditional methods to better characterize the risk of AMR in retail products.

## Introduction

Ground beef can be a reservoir for a variety of bacteria, including spoilage organisms and pathogenic foodborne bacteria. Multiple-hurdle intervention systems used during harvest and post-harvest are used to bolster meat safety and have shown to greatly reduce the bacterial load on carcasses and downstream products (Wheeler et al., 2014). However, even with ongoing improvements in food safety, it is estimated that foodborne pathogens caused 9.4 million episodes of foodborne illness, 55,961 hospitalizations and 1,351 deaths annually in the United States (Scallan et al., 2011). Though beef is not the most common source of foodborne outbreaks, retail ground beef products have been linked to multi-state outbreaks of foodborne illness and it is critical that we understand the bacterial community on and within retail beef products (Tauxe, 2006). Further, these bacteria can exhibit antimicrobial resistance (AMR) which is a public health concern as resistance in pathogens can lead to treatment failure in humans. Concerns regarding exposure to resistant bacteria or antimicrobial drugs (AMDs) in food have led to an increase in the practice of marketing animal products as being derived from animals that were “raised without antibiotics” (RWA). This is done to differentiate from products originating from conventional rearing systems (CONV) in which animals may have been treated with AMDs for control, prevention, or treatment of disease. This can lead consumers to believe that not using antibiotics is associated with decreased risk of exposure to AMR pathogens, but recent studies suggest minimal to no difference in AMR prevalence between these two types of production systems (Vikram et al., 2017, 2018, 2019). Due to animal welfare concerns related to infectious diseases, simply removing all AMD use from beef production is not as straightforward as consumers might believe (Karavolias et al., 2018). A limited body of research suggests that there is no significant difference in AMR in bacteria cultured from ground beef products labeled as CONV or RWA (LeJeune and Christie, 2004; Vikram et al., 2018), but little is known about AMR in the entire population of bacteria that can only be studied using culture-independent methods for characterizing resistance patterns.

Typically, studies of foodborne bacteria focus on individual pathogens or indicator organisms, such as *Escherichia coli*, and AMR is identified by phenotypic testing of isolates to an array of AMDs (Zhao et al., 2012). While these techniques provide insight into the AMR pattern for specific organisms, extrapolating these results to other bacterial species has not been validated and AMR found in these non-pathogens has not been shown to predict health risks in people. This is especially problematic given that AMR determinants can be transferred between pathogenic and non-pathogenic species. Alternatively, high throughput metagenomic sequencing can provide a holistic perspective on the community of microbial species (microbiome) and the profile of AMR genes they carry (resistome). Through the use of metagenomics, many environments and biological niches previously considered to have low or relatively simple bacterial biomass are now being re-discovered as having a complex microbiome (Berthelot et al., 2019; Castillo et al., 2019; Huebner et al., 2019; Stinson et al., 2019). However, using these techniques is accompanied by challenges in laboratory processing, particularly for food matrices where non-target DNA from the host organism (i.e., bovine DNA) is highly abundant. Thus, our research group has developed an approach to target-enrich AMR genes present in DNA samples using biotinylated RNA baits to increase the efficiency of sequencing the resistome. We have demonstrated biotinylated baits’ ability to increase “on-target” sequencing in cattle feces (Noyes et al., 2017). Here, we utilized the same approach to sequence the microbiome and resistome in commercial ground beef samples. The primary goal of this study was to characterize the microbiome and resistome of retail ground beef products with RWA label claims and conventional (CONV) retail ground beef products with no claims regarding antibiotics use. We also examined the within-sample resistome variability and whether the concentration of baits affected efficiency of target-enriched sequencing.

## Materials and Methods

### Study Design

Sixteen individually-packaged ground beef products were purposefully selected and purchased at 6 different retail grocery stores in Fort Collins, Colorado. Total DNA was extracted from each sample and subjected to 16S rRNA amplicon sequencing and target-enriched shotgun sequencing to characterize the microbiome and resistome, respectively. Biological replicates from each retail ground beef product were analyzed to assess within-sample variability and to test whether dilution of baits improved resistome sequencing performance.

### Sample Collection

Packages of ground beef (≥1 lb) were purchased from 6 different retail grocery stores in Fort Collins, Colorado on September 18, 2017 and stored at 4°C for 48 h until being further processed. Products were purchased with regard to production claims regarding exposure of animals to AMDs. Products with label claims for certified organic production or that otherwise specify the lack of AMD use during production were sampled as “Raised-Without Antibiotics” (RWA, *n* = 8) while products that did not have any label claims impacting AMD exposures of cattle were sampled as “conventional” products (CONV, *n* = 8). Products were sold in four different types of packaging: vacuum sealed, chub wrap, store grind and wrapped, or tray overwrap. Other metadata associated with each sample such as, packaging type and lean percentage, etc. were recorded (Supplementary File 1).

### Sample Processing and DNA Isolation

To replicate handling of retail ground beef products by typical consumers, samples were held at 4°C for 48 h before being opened. Packages were opened aseptically by first wiping the outside with 70% ethanol, followed by RNase AWAY (Thermo Fisher Scientific), and then cut open using a sterile, disposable scalpel. Ground beef (30 *g*) was removed aseptically from each package (*n* = 16) and placed in a new Filtra-Bag (VWR). An additional section of ground beef (30 *g*) was collected as biological replicates from each sample and was processed for target-enriched resistome sequencing using half diluted baits. All samples were homogenized using 100 ml of PBS and hand-massaged in a Filtra-Bag. Supernatant (15 mL) was then transferred to a sterile conical tube and centrifuged at 10,000 × *g* for 10 min. The resulting supernatant was discarded, and pellets were stored at −80°C until being processed for DNA extraction. A 950 μL aliquot of this pellet was used for total DNA isolation with the DNeasy PowerFecal Microbial Kit (Qiagen) according to manufacturer’s instructions. DNA was eluted in 50 μL of buffer solution and passed through the spin filter twice to optimize yield. DNA concentrations were measured with the Qubit dsDNA HS Assay Kit using the Qubit 2.0 Fluorometer according to manufacturer’s instructions (Thermo Fisher Scientific). If individual sample concentrations were <1 ng/μL, multiple extractions were pooled together to obtain this concentration.

### Library Preparation and Metagenomic Sequencing

DNA extracted from each sample (200–500 ng) was shipped to Novogene Corporation (Sacramento, CA, United States) for 16S rRNA gene amplicon sequencing to characterize the microbiome. The 292 bp V4 region of the 16S subunit was amplified with the 515F/806R primer set [5′-GTGCCAGCMGCCGCGG TAA-3′]/[5′-GGACTACHVGGGTWTCTAAT-3′]. Amplicon sequencing was performed on the Illumina HiSeq 2500 Sequencing System to produce paired end 250 bp reads (PE 250) at a targeted read depth of up to 100,000 PE reads per sample.

The SureSelectXT HS Reagent Kit for Illumina Paired-End Multiplexed Sequencing Library (Agilent Technologies) was used to prepare samples for target-enriched resistome sequencing. A customized bait design targeting AMR genes, “MEGaRICH” (Noyes et al., 2017), was used to improve “on-target” sequencing and reduce the challenge of sequencing microbial DNA from a sample predominantly containing host DNA. To gain further insight into bait performance, we included a biological replicate of each of the 16 packages of ground beef (2 × 30 *g* samples) that was processed with half diluted baits (32 total shotgun libraries) to assess if this improved on-target sequencing performance. Samples were transported to UC-Denver Genomics and Microarray Core Facility (Denver, CO, United States) and sequenced using the HiSEQ 4000 Sequencing System (Illumina) to produce paired-end 150 bp reads, targeting a read depth of 100 million PE reads per sample.

Summary statistics regarding the number of raw, trimmed, and non-host reads for each sample were compared using generalized linear models with the “glm” function and the R programming version 3.6 (R Development Core Team, 2008) to assess systematic bias across the following sequencing metadata: treatment, store, and dilution. Primary comparisons of interest were between CONV vs RWA sample labels and comparing sequencing results between typical vs diluted baits.

### Microbiome and Resistome Characterization

Details on all the commands used to analyze the data in this manuscript can be found at the project’s corresponding GitHub repository: https://github.com/meglab-metagenomics/Ground_beef_metagenomics_manuscript. To describe the profile of microbes present in ground beef products, reads from 16S rRNA amplicon sequencing were analyzed using the collection of tools contained in the Quantitative Insights Into Microbial Ecology version 2 software (Bolyen et al., 2019). Briefly, all reads are processed for sequence quality and denoising using DADA2 (Callahan et al., 2016). Taxonomic classification was performed using a naive bayes classifier trained on the GreenGenes database (McDonald et al., 2012), with chloroplast and mitochondrial DNA contaminants removed. Results were then exported into count tables and analyzed using the R statistical software.

To identify the resistome in ground beef products, the targeted AMR metagenomic sequencing samples were analyzed using the AmrPlusPlus 1.0 (AMR++) bioinformatic pipeline and the MEGARes resistance database v1.0.1 (Lakin et al., 2017). Further details on the pipeline can be found in the documentation website^1^. Briefly, read quality filtering is performed using Trimmomatic and host contamination is identified using the Burrows-Wheeler-Aligner (BWA) software with alignment to the Bos Taurus genome (Liu et al., 2009) and removal of corresponding reads with SamTools (Li et al., 2009). These non-host reads were then aligned to the MEGARes database (version 1.02) with BWA. Only gene accessions with reads aligning to >80% of the reference nucleotide sequence were considered for further analysis, with the exception of reads aligned to genes that require the presence of specific single nucleotide polymorphisms (SNPs) to confer resistance. These reads are identified, extracted from the corresponding samples, and re-classified separately using Resistance Gene Identifier (RGI; Alcock et al., 2020). We employed the “strict” classification threshold setting which incorporates detection models and CARD’s curated similarity cut-offs to increase accuracy in identifying functional AMR genes. To investigate the presence of AMR genetic determinants with high importance to public health when they are identified in human pathogens, a subset of AMR genes was selected *a priori* and data were searched to identify their presence: *bla*_*OXA*_, *bla*_*SME*_, *bla*_*IMI*_, *bla*_*NDM*,_ *bla*_*GES*_, *bla*_*KPC*_, *bla*_*CphA*_, *bla*_*TEM*_, *bla*_*SHV*_, *bla*_*CTX–M*_, *bla*_*CMY*_, *bla*_*OXA*_, *vga/vat*, and *cfr*.

### Count Matrix Processing

Differences in the microbiome and resistome between RWA and CONV ground beef were analyzed using the R programming software and complementary software packages. Everything required to replicate this analysis including count matrices, R environment, and R code, in addition to further descriptive figures can be found at this project’s GitHub repository^2^. Using “Binder 2.0” (Jupyter et al., 2018), an open source web service for sharing reproducible software environments, the results from this manuscript can also be explored interactively.

To account for differences in sequencing depth between samples, cumulative sum scaling (CSS) was used to normalize counts by using a scaling factor that reduces the influence of highly abundant taxa in sparse count tables (Paulson et al., 2013). The primary R packages used for count processing, normalization, and diversity analysis were “phyloseq,” “metagenomeSeq,” and “vegan” (McMurdie and Holmes, 2013; Paulson et al., 2013; Oksanen et al., 2014). The resistance data was then summarized to the class and mechanisms level to avoid bias at the “gene” level associated with irregular naming criteria for new resistance genes (Hall and Schwarz, 2016). For microbiome analysis, counts were taxonomically classified at 6 Linnaean levels: phylum, class, order, family, genus, and species, resulting in 6 microbiome count matrices for the microbiome. However, to reduce the repetitive reporting of results and because results at lower taxonomic levels are not as reliable (Caro-Quintero and Konstantinidis, 2012; Table 1), statistical results for microbiome are presented only at the phylum, class and order levels. In total, 5 unique normalized count matrices (i.e., 3 count matrices describing the microbiome and 2 count matrices characterizing the resistome) were analyzed and reported.

**TABLE 1:** Table of the percentage of 16S rRNA sequencing reads classified taxonomically to each Linnaean level, by treatment group (CONV vs RWA).

		Taxonomic rank					
Treatment group	Domain	Phylum	Class	Order	Family	Genus	Species
Raised without antibiotics (RWA)	100%	85.34%	85.34%	85.33%	81.70%	75.63%	73.51%
Conventional (CONV)	100%	89.88%	89.88%	89.87%	88.20%	82.83%	50.55%

### Statistical Analysis

Richness and Shannon’s diversity values were calculated for each sample using “vegan” and statistical comparisons were made using the “wilcox.test” function in R. Normalized counts were Hellinger-transformed for ordination using the metaMDS function from “vegan,” which employs non-metric multidimensional scaling on Euclidian distances. Analysis of similarities (ANOSIM) was used to test differences in the microbiome and resistome between CONV and RWA. Alternatively, to identify which specific features had significantly different numbers of alignments between treatment groups, metagenomeSeq’s “fitZig” function was used to fit a zero-inflated Gaussian (ZIG) model and compare log_2_-fold differences. The LIMMA package calculates the average log_2_-abundance (defined as “average expression”) for each feature across all samples in the ZIG model. LIMMA’s “makeContrast” function was then used for pairwise comparisons; *P*-values were adjusted for multiple tests using the Benjamini-Hochberg procedure. Alpha = 0.05 was selected as the threshold for statistical significance for all models. To account for spurious significant differences in low abundance features, only features with a log_2_-abundance >2 were considered.

## Results

### Sequencing Results

Sequencing of the 32 samples processed with AMR target-enrichment produced >1.3 billion paired end reads (mean: 42.6M, range: 8.5M–67M). Read quality filtering removed an average 3.7% of raw reads from each sample (range: 3.4–4.1%), with the majority of reads removed from each sample after removal of bovine host DNA sequences (mean: 99.43%, range: 96.8–99.95%). There was a difference (*P* < 0.05) in the number of raw reads produced for the bait capture sequencing between CONV and RWA samples, but this was likely influenced by a lower abundance (*P* < 0.05) of reads in samples from vacuum sealed packing type. On average, samples from chubs had 51.3 million reads per sample compared to 36.9 million reads per sample in vacuum packaging (Supplementary File 1). With 16S rRNA amplicon sequencing, >3.1 million paired end reads were produced (mean: 194,408.3 reads per sample, range: 100,939–219,822). Filtering to improve overall read quality removed, on average, 7.3% of raw reads from each sample (range: 4.7–12.38%). There was no difference (*P* > 0.05) in the number of 16S amplicon sequencing reads between the CONV and RWA samples or by packaging type.

### Resistome Results

Following alignment of reads to the MEGARes database, removal of duplicate reads, and re-classification of reads aligning to gene accessions requiring SNP confirmation with RGI, a total of 267,922 alignments to AMR gene accessions (“hits”) were identified across all samples (mean: 8,372 per sample, range: 80–51,868). Hits were classified to 565 different gene accessions, which represented genes that confer resistance to 17 different drug classes through 32 resistance mechanisms (Supplementary File 2). Gene accessions classified using CARD’s RGI tool can require the presence of specific SNPs; further details can be examined using CARD’s ontology browser^3^. There were no differences (*P* > 0.05) in the resistome composition between biological replicates, suggesting that microbial populations were relatively evenly distributed in purchased products (Supplementary Figure 1). Further, there was no differences (*P* > 0.05) in the total number of AMR alignments, richness, or Shannon’s diversity when using standard, or half-concentrations of baits.

**FIGURE 1:**
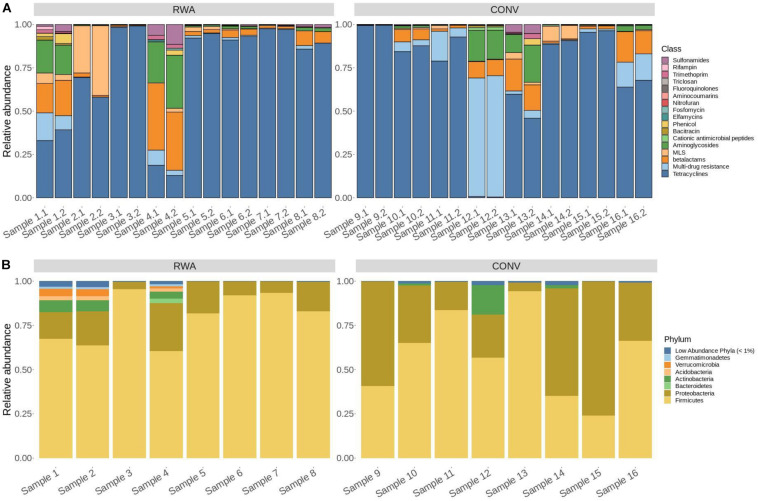
Stacked bar graphs showing the **(A)** resistome composition and **(B)** microbiome composition of ground beef samples in this study. Individual ground beef samples are on the *x*-axis and relative abundance proportions are on the *y*-axis. **(A)** Resistome composition at the AMR drug class level for all 16 CONV and RWA ground beef samples in the study, including biological replicates (*N* = 32). **(B)** Microbiome composition at the phylum level for all 16 ground beef samples, with phyla composing less than 1% of all microbiome counts labeled as “Low Abundance Phyla.”

Out of hits classified to 17 AMR drug classes, alignments to tetracycline resistance gene accessions were predominant and made up 91.8% of normalized hits across all samples. The next most abundant hits aligned to the drug class, macrolide-lincosamide-streptogramin (MLS; 3.4%), multi-drug resistance (2.3%), and betalactams (1.4%) with the remaining 13 classes each comprising less than 1% of counts. Of these hits to gene accessions that confer tetracycline resistance, a majority were classified as encoding tetracycline resistance ribosomal protection proteins and major facilitator superfamily (MFS) efflux pumps (61% and 39%, respectively). This pattern of relative abundance for resistome composition was generally consistent across samples (Figure 1).

**FIGURE 2:**
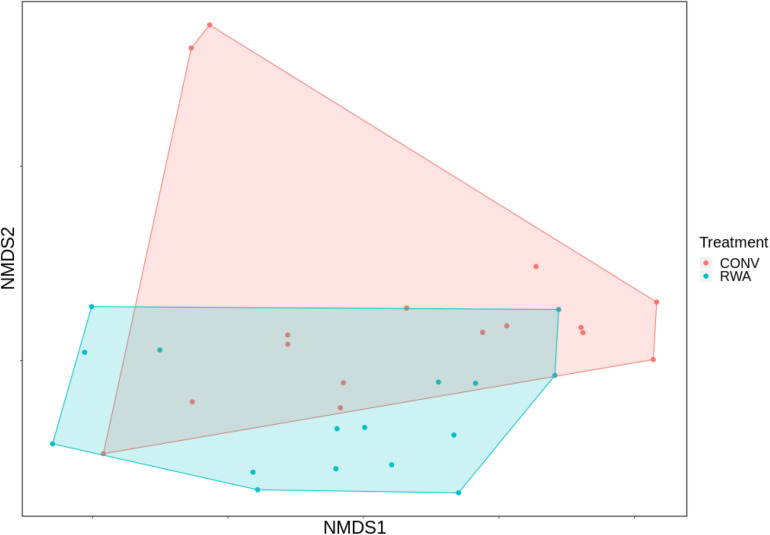
Ordination comparing resistome composition at the AMR drug class level, using non-metric multidimensional scaling (NMDS), between labeling types on ground beef products; conventional (CONV) vs raised without antibiotics (RWA).

**FIGURE 3:**
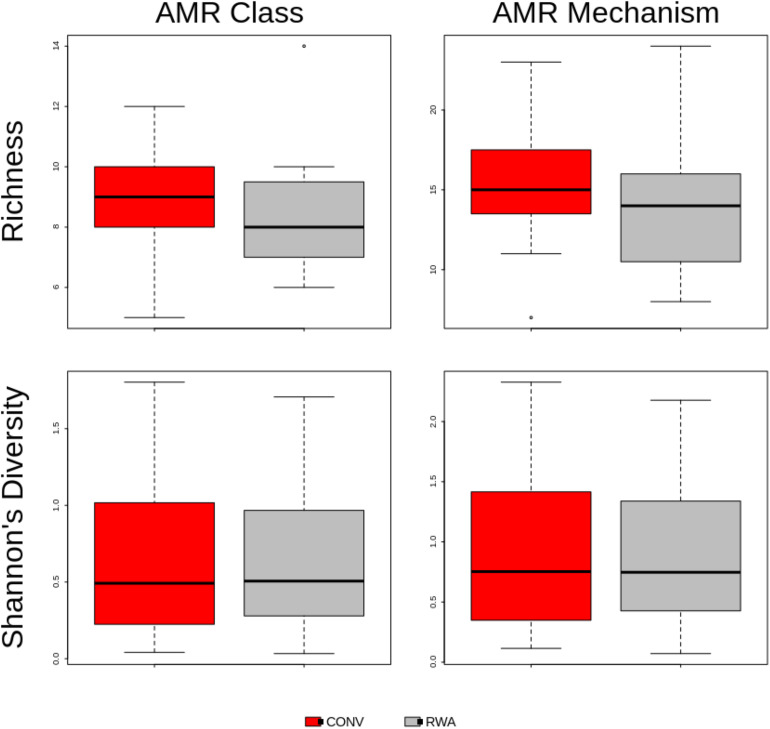
Boxplot of resistome richness and Shannon’s diversity at the AMR class and mechanism levels of the two study groups, CONV vs RWA. The horizontal line is the median value, the middle box indicates the inter-quantile range, whiskers represent values within 1.5 IQR of the lower and upper quartiles, and individual points show outlier values.

At the AMR class level, ANOSIM testing suggested that the overall resistome composition did not differ (*R* = 0.001, *P* > 0.05) between ground beef label types (Figure 2). However, at the mechanism level there was a significant separation between resistomes by label type (*R* = 0.13, *P* < 0.05), though the small *R* value suggests this was not a large difference (Supplementary Figure 2). Shannon’s diversity index comparisons were not different (*P* > 0.05) between CONV and RWA samples at the class and mechanism levels, but richness was only significantly different at the mechanism level (*P* < 0.05; Figure 3). On the other hand, resistome composition at the mechanism level clustered significantly based on the type of sample packaging (*R* = 0.32, *P* < 0.05). Further, resistome composition clustered by source retail store at the class (*R* = 0.29, *P* < 0.05) and mechanism levels (*R* = 0.46, *P* < 0.05; Supplementary File 3). In combination, these results suggest that the differences observed between resistome composition in our ground beef samples could be driven by something other than CONV versus RWA labels.

The ZIG model identified that out of 11 drug classes, six were higher (*P* < 0.05) in CONV than RWA samples. While there were no differences in tetracycline resistance, which made up >90% of all alignments, the CONV ground beef samples had a significantly higher relative abundance of multi-drug resistance, betalactams, cationic antimicrobial peptides, and elfamycin (*P*-value < 0.05), whereas alignments to trimethoprim and phenicol resistance were more abundant (*P* < 0.05) in RWA products (Supplementary File 4). At the mechanism level, alignments to tetracycline resistance MFS efflux pumps were higher (*P* < 0.05) in CONV samples compared to RWA. Differences in the differential abundance of specific mechanisms mirrored differences at the class level. Of 16 AMR mechanisms with an average expression >2, 10 were significantly different between CONV and RWA samples (Supplementary File 4). Of the list of genes identified *a priori* as being important to medicine and public health, *bla*_*OXA*_, *bla*_*SME*_, *bla*_*IMI*_, *bla*_*CphA*_, *bla*_*TEM*_, *bla*_*CTX–M*_, *bla*_*CMY*_, *bla*_*OXA*_, and *vga/vat* were identified in samples. Overall, these genes were sparsely represented and in total accounted for only 3,439 CSS normalized counts (1.2% of the resistome) across all 32 samples with *bla*_*TEM*_ and *bla*_*CTX–M*_ genes making up more than half of those counts (Supplementary Figure 3).

### Microbiome Results

A total of 2,496,913 reads were classified taxonomically with an average of 156,057.1 reads per sample (range: 87,476–191,521). In all, 4949 amplicon sequence variants (ASVs) were identified with DADA2 and together represented 43 phyla, 119 classes, and 201 orders (Supplementary File 5). Two phyla, Firmicutes and Proteobacteria, predominated in the microbiome in this study and together accounted for >98% of all normalized counts (69.6% and 29.0%, respectively; Figure 1). These phyla were each comprised of a single class that contributed a majority of counts in each taxa. The Bacilli class represented 98.8% of alignments in the Firmicutes phylum, and the Gammaproteobacteria class comprised 81.3% of Proteobacteria across all samples. At the order level, Lactobacillales (68.5%), Vibrionales (19.3%), Neisseriales (4.78%), Enterobacteriales (2.54%), and Pseudomonadales (1.46%) were the most abundant taxa, with the remaining taxa making up less than 1% of classified reads. While the relative abundances differ, the taxa making up the microbiome samples in this study largely resemble the results from metagenomic studies of beef carcass trimmings and ground beef samples (Stellato et al., 2016; Weinroth et al., 2019; Figure 1). At the genus level, common spoilage taxa like Lactococcus, Leuconostoc, and Lactobacillus were also identified in the majority of ground beef samples (Borch et al., 1996; Supplementary Figure 4).

**FIGURE 4:**
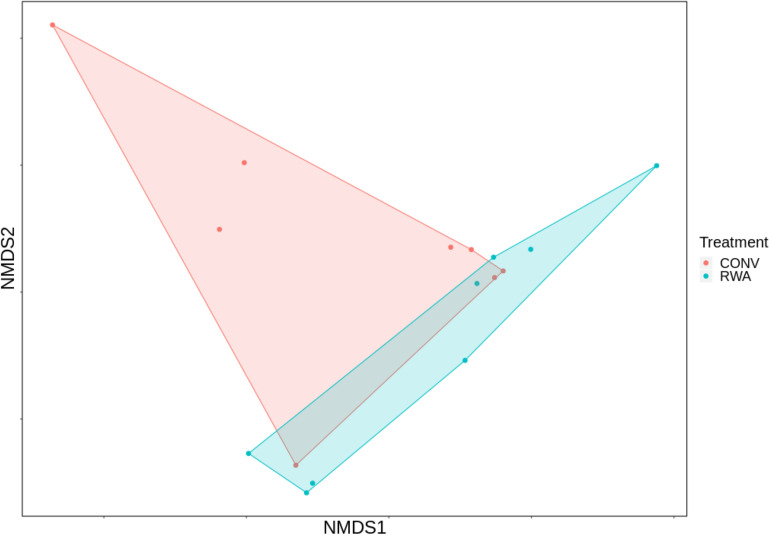
Ordination comparing microbiome composition at the order taxonomic level, using non-metric multidimensional scaling (NMDS), between labeling types on ground beef products; conventional (CONV) vs raised without antibiotics (RWA).

**FIGURE 5:**
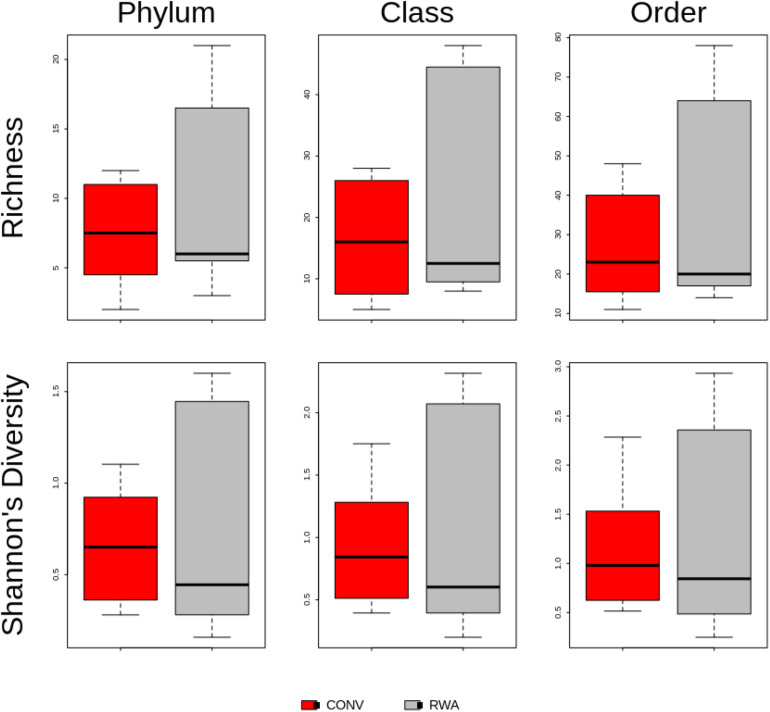
Boxplot of resistome richness and Shannon’s diversity at the taxonomic phylum, class and order levels of the two study groups, CONV vs RWA. The horizontal line is the median value, the middle box indicates the inter-quantile range, whiskers represent values within 1.5 IQR of the lower and upper quartiles, and individual points show outlier values.

Overall, significant differences were observed between microbiomes found in CONV and RWA beef products at the class (*R* = 0.18, *P* < 0.05) and order levels (*R* = 0.17, *P* < 0.05; Figure 4). However, there were no significant differences in richness or Shannon’s diversity index between label type (Figure 5). Out of 43 phyla, only 3 were different (*P* < 0.05) in relative abundance, with CONV samples containing increased proportions of Proteobacteria and decreased Planctomycetes and Crenarchaeota compared to RWA samples (Supplementary File 6). Out of 11 classes that were differentially abundant between treatment groups, Gammaproteobacteria and Clostridia were found in higher relative abundances in CONV samples with the other 9 classes found in lower abundance in RWA samples (Supplementary File 6). Indeed, like in the resistome ordination, samples from different retail stores were significantly clustered at the phylum (*R* = 0.24, *P* < 0.05), class (*R* = 0.32, *P* < 0.05), and order (*R* = 0.29, *P* < 0.05) levels (Supplementary Figure 5). Microbiome composition also clustered with greater *R* values based on sample packaging type, at the phylum (*R* = 0.37, *P* < 0.05), class (*R* = 0.37, *P* < 0.05), and order (*R* = 0.4, *P* < 0.05) levels.

## Discussion

Our results suggest that the overall resistome composition of retail ground beef products with RWA packaging labels do not differ from products not carrying claims regarding AMD exposures during cattle production (Figures 2, 3). This research adds to the growing body of literature suggesting that AMD use in livestock production does not have a strong impact, compared to other factors, on the metagenomic resistance of bacterial populations on beef products (Vikram et al., 2017, 2018; Weinroth et al., 2018). Further investigations of retail meat products using metagnomic sequencing are warranted to substantiate these conclusions. AMDs are used in animal populations to control, prevent, and treat bacterial diseases. Systematically restricting drug use can affect animal health and well-being and these issues must be addressed with balanced consideration regarding the production of meat products that are sold with the “Raised Without Antibiotics” label claim (Singer et al., 2019).

The novel target-enriched resistome sequencing employed in this study was essential to the success of these investigations, especially considering the low microbial abundance and high amounts of host (off-target) DNA. Previous investigations of metagenomic sequencing of feces suggested that our system of target-enriched sequencing created an average of 4x increase in on-target sequencing depth among common taxa (i.e., those with relative abundance >2; Noyes et al., 2017). A previous investigation of resistome ecology in beef production systems did not identify any reads aligning to AMR determinants using shotgun sequencing of beef rinsates with an average sequencing depth of 6 × 10^7^ reads per sample (Noyes et al., 2016). Together, these previous studies suggest that depth of traditional shotgun sequencing would need to be increased to approximately >6 × 10^11^ reads per sample to provide a similar ability to investigate the resistome of beef products as demonstrated herein. Despite this target enrichment, the relatively low numbers of hits in these samples reaffirms the low microbial biomass that can be found in retail beef products in the United States. While target-enriched sequencing provides significant opportunities for resistome investigations, the library preparation costs are higher. Consultation with scientists from the manufacturer led to a hypothesis that target enrichment in ground beef samples may be improved by reducing the concentration of baits used in the library preparation. Results comparing technical replicate samples prepared with full- and half-concentration baits were not significantly different (Figure 1 and Supplementary Figure 1), suggesting that the lower concentration can provide greater efficiency when analyzing microbial populations in samples such as ground beef.

To date, there are no other published comparisons of RWA and CONV ground beef products using metagenomic resistome sequencing, but culture-based investigations of beef have found limited differences in abundance of specific target bacteria and resistance genes between the products with different label claims regarding AMD exposures (Vikram et al., 2018). The resistome of ground beef samples included in this study was predominated by alignments to gene accessions conferring resistance to tetracycline drugs. These results are consistent with other studies of cattle environments, using both aerobic culture of *Salmonella enterica* and *Campylobacter* spp. as well as recent studies using metagenomic sequencing, which also report tetracycline resistance as one of the most common classes of resistance identified in these samples (Zhao et al., 2006, 2010; Noyes et al., 2016; Doster et al., 2018; Weinroth et al., 2018; Huebner et al., 2019). This is logical, considering that a large portion of the microbiome of ground beef products is generally considered to originate from fecal and environmental material found on animal hides at the time of slaughter (Bacon et al., 2000). It is also important to note that AMR is an ancient phenomena and that resistance to certain drug types, including the tetracycline and betalactam classes of antimicrobial compounds, have been identified in “pristine environments” suggesting the presence of these elements prior to the use of AMDs in livestock production (D’Costa et al., 2011; Perron et al., 2015).

A limited number of other studies have investigated the microbiome of meat products using 16S amplicon sequencing, and these also showed a predominance of Firmicutes and Proteobacteria phyla in beef samples (Poirier et al., 2018; Weinroth et al., 2019), as have studies investigating the microbiome of chicken and pork samples (Poirier et al., 2018; Lauritsen et al., 2019). Interestingly, Photobacterium, a genus only recently described as being associated with meat products was identified making up more than 50% of the microbiome in 3 out of 4 CONV samples in chub packaging (Supplementary Figure 4; Hilgarth et al., 2018).

While there was no significant separation between the overall resistome composition at the AMR class level for products with different AMR exposure claims (Figure 2), there were some significant differences between RWA and CONV products for individual taxa of interest. For example, CONV samples had a significantly higher relative abundance of the mechanism, tetracycline resistance MFS efflux pump despite there being no significant differences in the relative abundance of hits that mapped to the higher taxonomical level of resistance to tetracyclines as a drug class. This suggests that phenotypic testing of AMR and metagenomic analysis at the broad classification of drug class could miss differences that become apparent when analyzing data at lower taxonomic levels (e.g., mechanism or gene group) using sequence-based approaches. This has implications with regard to the potential for selection of specific genes or bacterial clones as a result of AMD exposures and should be further explored. Further, this must be balanced with the limitation that by describing the DNA in a sample, metagenomic sequencing only reports the “potential” resistome function in a microbial population and cannot yet be directly linked with phenotypic function in pathogenic bacteria. Understanding the effects of AMD use in the context of beef production systems requires that these results be carefully interpreted in order to appropriately evaluate risks to human and animal health.

The ground beef samples in this study were purchased from 6 different retail stores to provide a diversely representative source of products, however, this study was not designed to test the effect of handling at the store. Nonetheless, our results suggest that the handling of ground beef products by the retail store, could potentially influence the microbiome and resistome of retail ground beef products. Further, the type of packaging was associated with greater differences in the microbiome as we observed an overabundance of the bacterial phyla, Proteobacteria, in chub packaging compared to other packaging types (Supplementary Figure 4), but the resistome composition did not appear to be distinct in chub packaging samples (Supplementary Figure 5). There were also no major differences in the resistomes of replicate samples obtained from the same packages. These findings suggest that microbial populations are homogenized and relatively evenly distributed within single batches of ground beef, as might be expected from production methods. However, further comparisons within and between regions are needed to further evaluate the expected variation in microbiome and resistome composition. Future investigations are also warranted regarding the effects of different product handling protocols (e.g., holding temperatures, product age, packaging, product handling before and after arriving at retail stores, etc.) to identify possible drivers of microbiome changes.

Metagenomic sequencing is a promising tool for characterizing the microbiome and resistome in retail ground beef products and has potential to be used for tracing individual sequence variants through the food chain. Nonetheless, innovative methods are needed to reduce sequencing cost and improve sequencing depth to get a more detailed perspective of the resistome on ground beef. Importantly, results from metagenomic sequencing must be carefully considered in parallel with traditional methods to better characterize the risk of AMR in retail products.

## Data Availability Statement

The datasets generated for this study can be found in the BioProject number PRJNA608954.

## Author Contributions

KB, PM, TW, JS, and TA participated in and provided oversight of all aspects of the study including securing funding, design, laboratory procedures, data analysis, and report preparation. KB and PM contributed equally as senior authors. ED participated in study conduct, performed bioinformatic analysis, and drafted the manuscript. KT, MW, KB, JS, and TA participated in study conduct and collected study samples. JP and KC participated in study conduct and laboratory analyses. All authors read, edited, and approved the final manuscript.

## Conflict of Interest

The authors declare that the research was conducted in the absence of any commercial or financial relationships that could be construed as a potential conflict of interest.

## Footnotes

1 http://megares.meglab.org/amrplusplus

2 https://github.com/meglab-metagenomics/Ground_beef_metagenomics_manuscript

3 https://card.mcmaster.ca/browse

## Supplementary Material

Supplementary Figure 1 Ordination comparing resistome composition at the AMR drug class level, using non-metric multidimensional scaling (NMDS), between biological replicates from the same ground beef product are grouped by color.

Supplementary Figure 2 Ordination comparing resistome composition at the AMR mechanism level, using non-metric multidimensional scaling (NMDS), between CONV and RWA samples.

Supplementary Figure 3 Heatmap of the number of samples with counts for “clinically important genes” on the *y*-axis, by sample packaging type on the *x*-axis.

Supplementary Figure 4 Stacked bar graph of the microbiome composition at the genus level with samples on the *x*-axis and relative abundance on the *y*-axis, by packaging type.

Supplementary Figure 5 NMDS ordination of the microbiome by retail store at all levels.

Supplementary Figure 6 Stacked bar graph of the microbiome composition at the phylum level with samples on the *x*-axis and relative abundance on the *y*-axis, by packaging type.

Supplementary Figure 7 Stacked bar graph of the resistome composition at the class level with samples on the *x*-axis and relative abundance on the *y*-axis, by packaging type.

Supplementary File 1 Excel spreadsheet containing metadata for the 16 microbiome samples and 32 resistome samples.

Supplementary File 2 Excel spreadsheet containing the resistome raw count matrix.

Supplementary File 3 Excel spreadsheet containing results for ANOSIM and Wilcoxon statistical comparisons between sample groups.

Supplementary File 4 Excel spreadsheet containing results from differential resistome feature abundance testing using a zero-inflated Gaussian (ZIG) model.

Supplementary File 5 Excel spreadsheet containing the microbiome raw count matrix.

Supplementary File 6 Excel spreadsheet containing results from differential microbiome feature abundance testing using a zero-inflated Gaussian (ZIG) model.
