# Supplementary figures and images for: Metagenomic Characterization of the Microbiome and Resistome of Retail Ground Beef Products
Source: Front Microbiol. 2020 Nov 6;11:541972. doi: 10.3389/fmicb.2020.541972 (PMC7677504; doi:10.3389/fmicb.2020.541972)

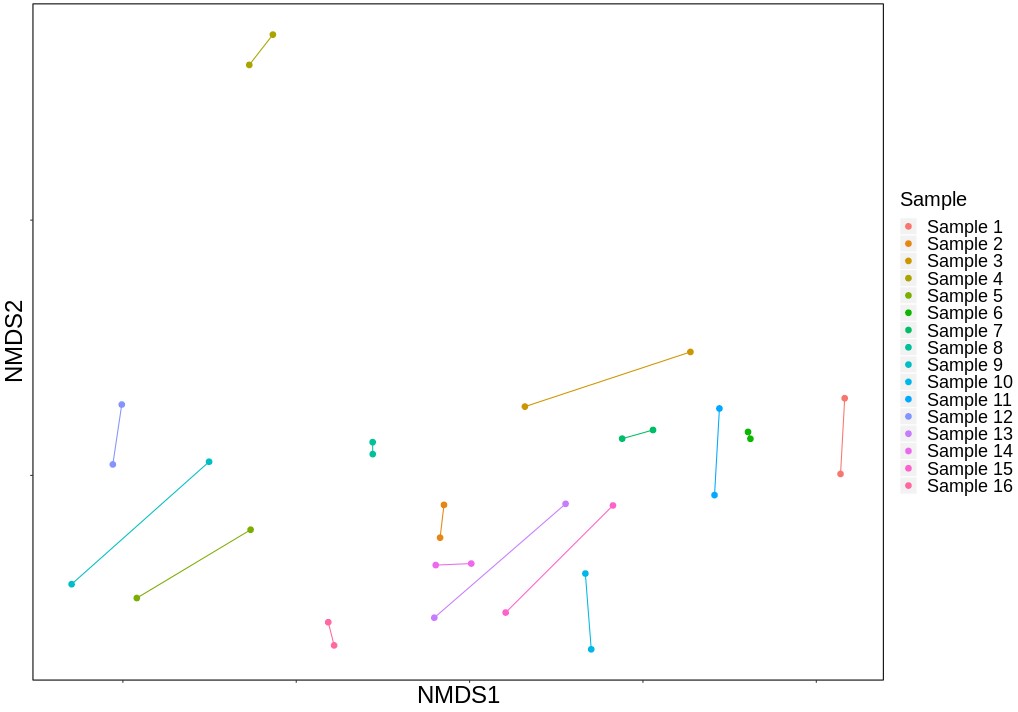

Supplement: Supplementary Figure 1 — Ordination comparing resistome composition at the AMR drug class level, using non-metric multidimensional scaling (NMDS), between biological replicates from the same ground beef product are grouped by color. [file Image_1.JPEG]

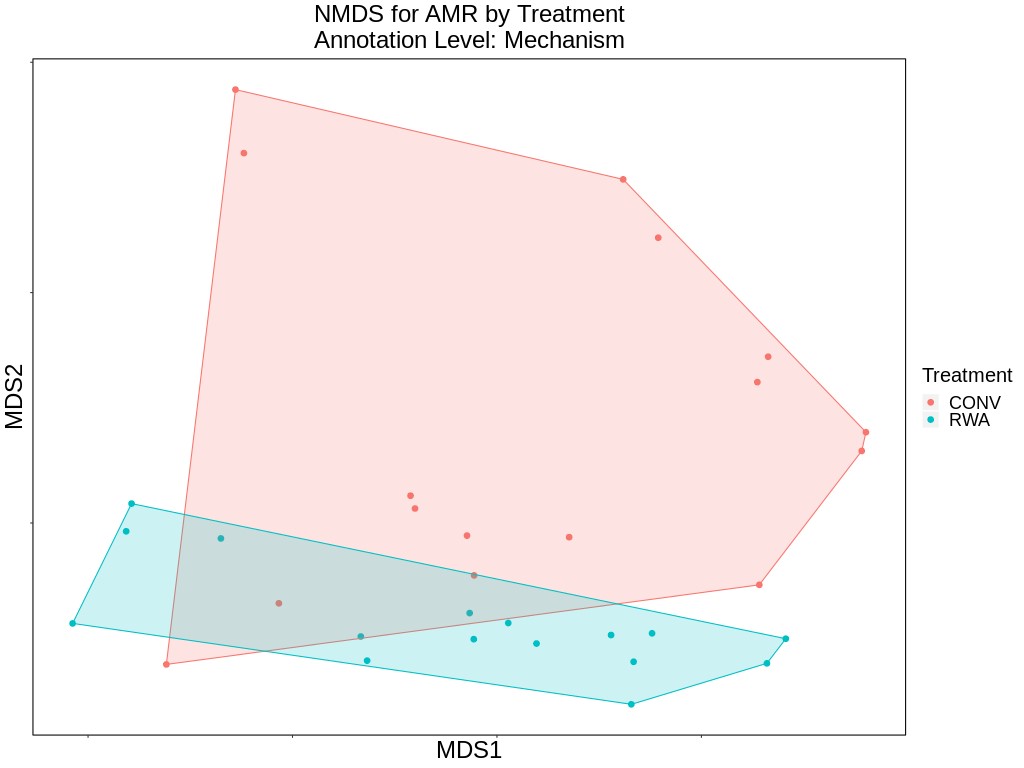

Supplement: Supplementary Figure 2 — Ordination comparing resistome composition at the AMR mechanism level, using non-metric multidimensional scaling (NMDS), between CONV and RWA samples. [file Image_2.JPEG]

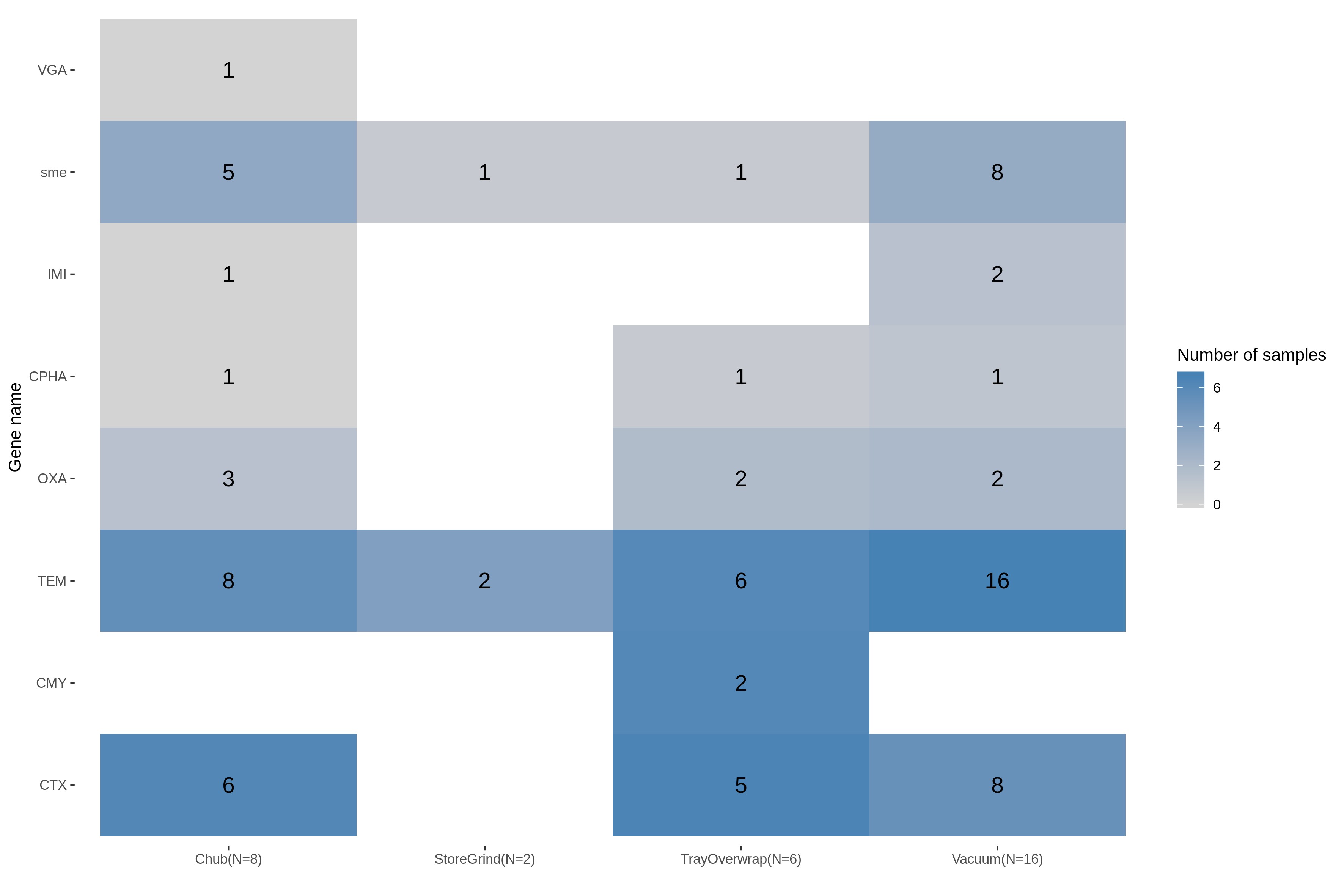

Supplement: Supplementary Figure 3 — Heatmap of the number of samples with counts for “clinically important genes” on the y-axis, by sample packaging type on the x-axis. [file Image_3.JPEG]

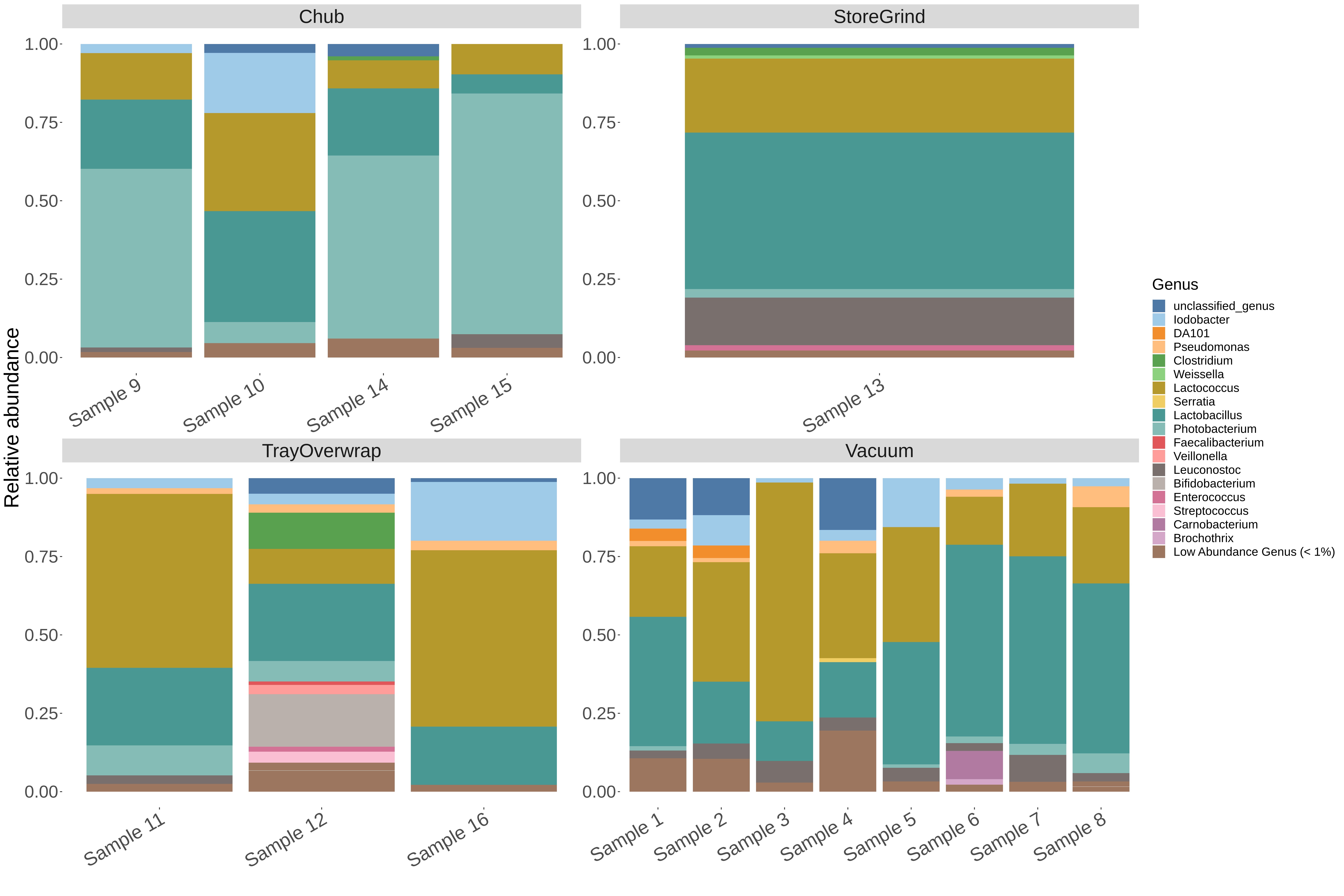

Supplement: Supplementary Figure 4 — Stacked bar graph of the microbiome composition at the genus level with samples on the x-axis and relative abundance on the y-axis, by packaging type. [file Image_4.JPEG]

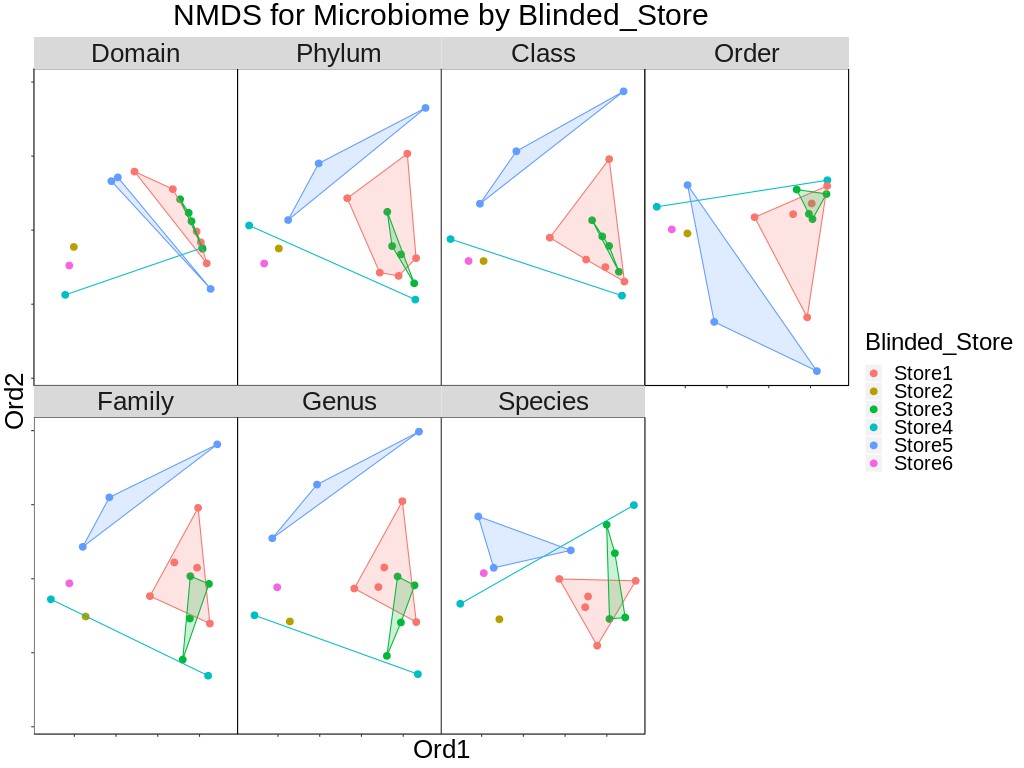

Supplement: Supplementary Figure 5 — NMDS ordination of the microbiome by retail store at all levels. [file Image_5.JPEG]

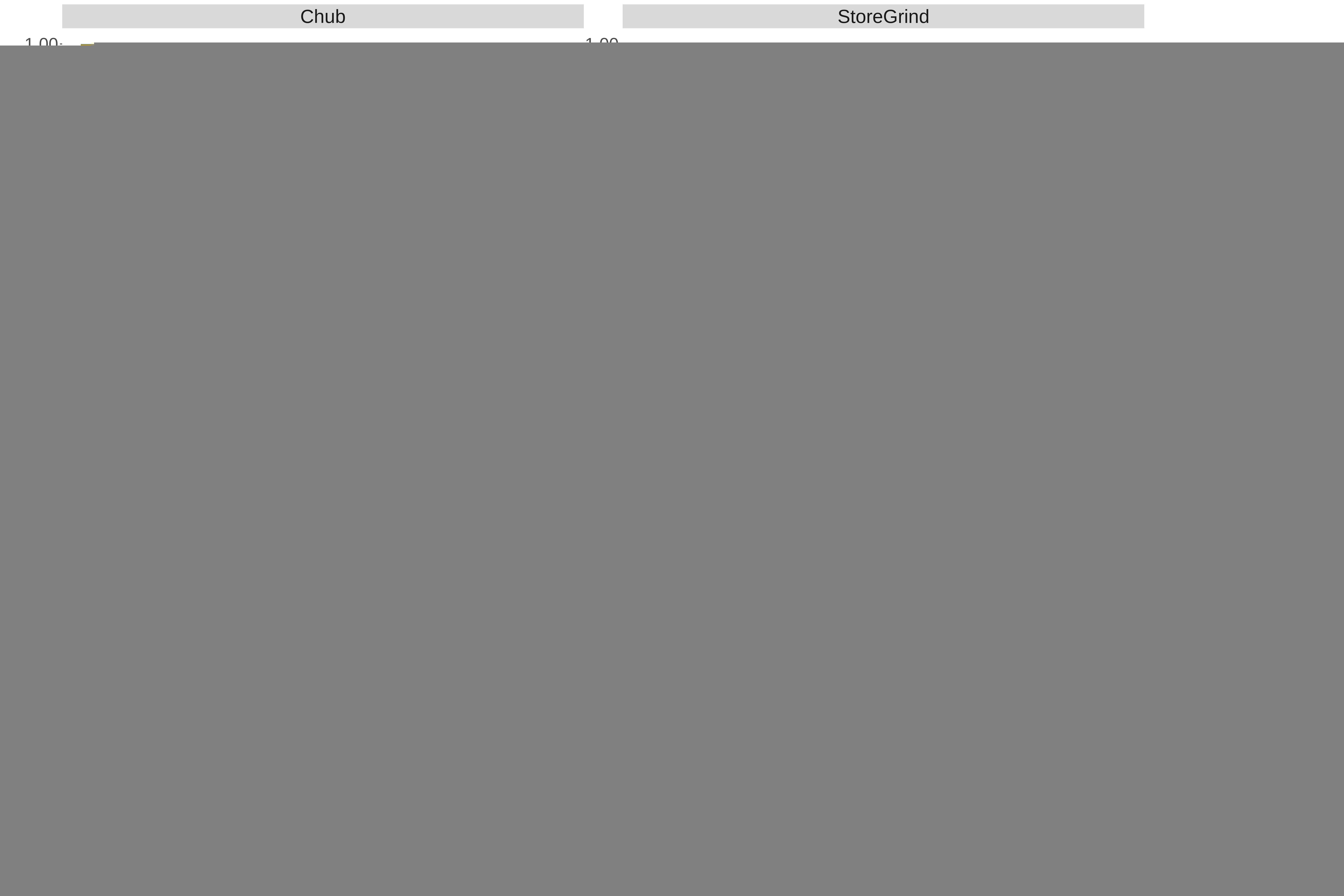

Supplement: Supplementary Figure 6 — Stacked bar graph of the microbiome composition at the phylum level with samples on the x-axis and relative abundance on the y-axis, by packaging type. [file Image_6.JPEG]

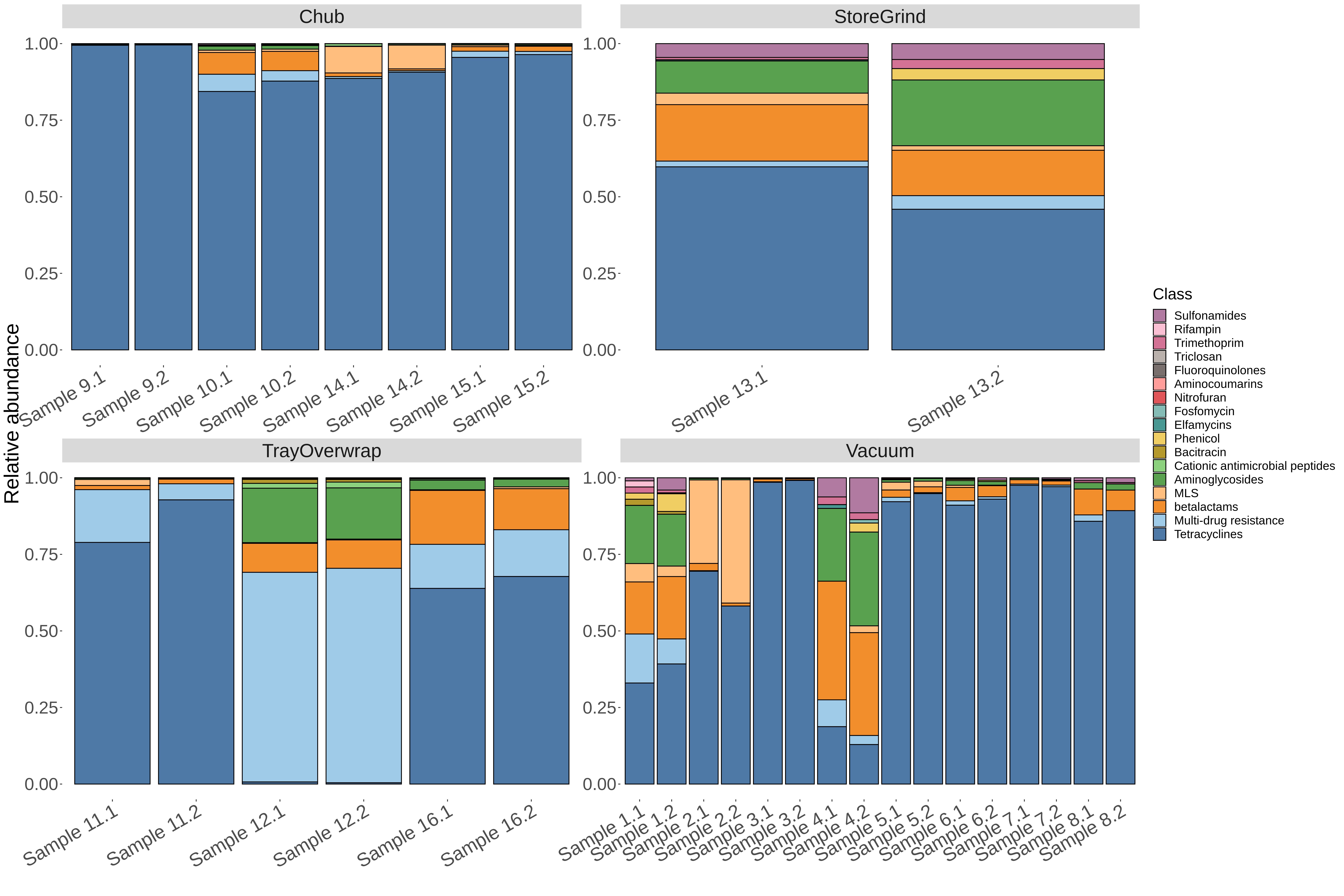

Supplement: Supplementary Figure 7 — Stacked bar graph of the resistome composition at the class level with samples on the x-axis and relative abundance on the y-axis, by packaging type. [file Image_7.JPEG]
